# Supplementary material for: Digital droplet PCR accurately quantifies SARS-CoV-2 viral load from crude lysate without nucleic acid purification
Source: Sci Rep. 2021 Jan 12;11:780. doi: 10.1038/s41598-020-80715-1 (PMC7804156; doi:10.1038/s41598-020-80715-1)
Supplement: Supplementary file 1 — Supplementary Information. [file 41598_2020_80715_MOESM1_ESM.docx]

Title: Digital droplet PCR accurately quantifies SARS-CoV-2 viral load from crude lysate without nucleic acid purification

**Authors:** Harish N. Vasudevan,^a,b,*^ Peng Xu,^a,*^ Venice Servellita,^c,d^ Steve Miller,^c^ Leqian Liu,^a^ Allan Gopez,^c^ Charles Y. Chiu,^c,d,e^ Adam R. Abate^a,f,#^

**Affiliations**

^a^Department of Bioengineering and Therapeutic Sciences, University of California San Francisco, CA, USA

^b^Department of Radiation Oncology, University of California San Francisco, CA, USA

^c^Department of Laboratory Medicine, University of California San Francisco, CA, USA

^d^UCSF-Abbott Viral ﻿Diagnostics and Discovery Center, San Francisco, CA, USA

^e^Department of Medicine, Division of Infectious Diseases, University of California San Francisco, CA, USA

^f^Chan Zuckerberg Biohub, San Francisco, California, USA

* These authors contributed equally to this study.

**Corresponding Author**

^#^Adam R. Abate, Ph.D.

Department of Bioengineering and Therapeutic Sciences, University of California, San Francisco, 1700 4^th^ St, San Francisco, California, USA

Email: [adam@abatelab.org](mailto:adam@abatelab.org)

**Supplementary Figures.**

**
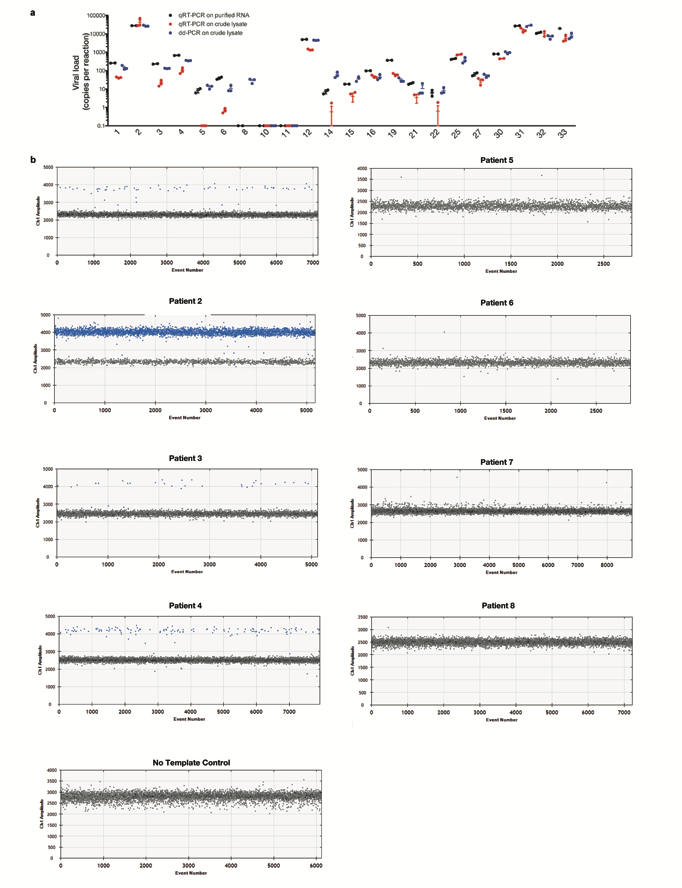
**

**Supplemental Figure 1.** (a) Fluorescence intensities as measured by BioRad QX200 Droplet Reader for example clinical nasopharyngeal patient samples 1-8 and a SeraCare no template control. (b) Absolute quantification by ddPCR from crude lysate show strong concordance with qRT-PCR from purified RNA in contrast to qRT-PCR from crude lysate (n=22 samples).
